# Value of multidetector computed tomography in the diagnosis of mucosa-associated lymphoid tissue-lymphomas in the parotid gland: A case report and review of the literature

**DOI:** 10.3892/ol.2013.1769

**Published:** 2013-12-20

**Authors:** YUE DONG, FENG WEN, AIJUN SHI, HONG WEI GUAN, YING GE, YUAN JIANG

**Affiliations:** 1 Department of Radiology, The First Affiliated Hospital of Dalian Medical University, Dalian, P.R. China; 2 Department of Radiology, Shengjing Hospital, China Medical University, Shenyang, Liaoning, P.R. China

**Keywords:** parotid gland, mucosa-associated lymphoid tissue lymphomas, computer tomography, enhancement

## Abstract

The present study aimed to review the multidetector computed tomography (MDCT) imaging features of eight mucosa-associated lymphoid tissue (MALT)-lymphoma cases of the parotid gland and to explore the diagnostic value of MDCT. A total of eight patients with pathologically confirmed MALT-lymphomas of the parotid gland underwent pre-operative MDCT plain and dual-phase scans. The changes in the CT values and enhancement patterns of the tumors were assessed. Quantitative analysis was performed to determine the CT value changes of the tumors in the various enhanced phases compared with the plain scan. The MALT-lymphomas of the parotid gland exhibited even density isodense or hyperdense nodules, with occasional calcification and necrosis. The dual-phase scan of the MALT-lymphomas revealed a pattern of lower or moderate enhancement, circumambient enhancement or delayed enhancement. The MALT-lymphomas were closely associated with Sjögre’s syndrome and demonstrated malignant features and isodense or hyperdense nodules and lower or moderate enhancement on the CT scans.

## Introduction

Mucosa-associated lymphoid tissue (MALT)-lymphoma is a distinctive subtype of non-Hodgkin lymphoma, whose concept was first put forward by British pathologists, Isaacson and Wright (1). MALT-lymphoma frequently occurs in the gastrointestinal tract and rarely occurs in the salivary gland. The condition is difficult to diagnose using imaging. Studies have previously reported the imaging features of gastric and pulmonary MALT-lymphoma (2,3). However, imaging studies of MALT-lymphomas of the parotid gland have not yet been published. The present study reviewed and analyzed the clinical, computed tomography (CT) imaging and pathological features of eight cases of pathologically confirmed MALT-lymphomas of the parotid gland in order to improve the imaging and clinical diagnosis.

## Case report

### Clinical data

A total of eight patients, one male and 7 females, with pathologically confirmed MALT-lymphomas were analyzed between January 2005 and November 2011. The age range of the patients was 14–80 years, with a median age of 52 years and a mean age of 50.9 years. Written informed consent was obtained from the patients.

### Methods

All the patients underwent spiral multidetector CT (MDCT) plain and enhancement scans of the parotid gland using the GE Lightspeed 16-row MDCT machine (GE Healthcare, Fairfield, CT, USA). The plain scan covered the area between the hyoid bone and the base of the skull using the following parameters: Column voltage, 120 KV; column electric current, 240–260 mAs; slice thickness, 2.5 mm; interval 0; and matrix, 512×512. A high-pressure syringe was used to inject 300 mg/ml Ultravist (Shanghai Boyier Chemical Co., Ltd., Shanghai, China) into the anterior middle vein of the elbow at a dosage of 50 ml and with an injecting speed of 3.5 ml/sec. A dual-phase scan was also performed. The arterial phase of the scan began at 25 sec and the venous phase at 75 sec into the scan. The slice thickness was 2.5 mm, with a 512×512 matrix. A standard algorithm was adopted for the reconstruction. Follow-up examinations were performed in all eight cases.

### Immunohistochemistry

Deparaffinized sections of patient tissue samples were washed with phosphate-buffered saline (PBS) three times for 5 min each. In order to block the endogenous peroxidase activity, the sections were dipped in PBS that contained 3% H_2_O_2_ for 10 min, then were immersed with 10% sheep serum for 30 min and incubated overnight with the specific antibodies, anti-CD3, CD5, CD19, CD20, CD43 and CD79a (Abcam, Cambridge, UK). The sections were washed and stained according to the manufacturer’s instructions.

### Imaging data

All the CT images of the patients were analyzed by two experienced radiologists in the Picture Archiving and Communication System diagnostic terminal of the Shanghai Daijia corporation (Shanghai, China) in order to assess the pathological position, number, morphology, edge, size, density and dual-phase enhancement features of the lymphomas. Various outcomes were agreed upon following negotiation. The size was determined by measuring the two largest diameters of the tumor and adopting the mean value. The plain CT scan values were measured and the CT enhancement features were assessed to see whether the enhancement was uniform and to analyze the increased value of the dual-phase enhancement CT compared with the plain scan. The CT value was from the target area of tumor parenchyma ~2 mm in diameter. This was measured three times and the mean value was calculated.

### Clinical data

As shown in Table I, all the patients presented with painless nodules in the parotid gland and the course of the disease varied between 20 days and six years. Of the total cases, seven included symptoms of a dry mouth and dry eyes, which is similar to the symptoms of Sjögre’s syndrome. The patients from cases 1 and 3 felt slight pain when the affected area was palpated and case 4 demonstrated symptoms of facial nerve involvement. The nodule of case 2 had invaded into the deep lobe of the parotid gland and linked with the sternocleidomastoid muscles, and the nodule of case 6 was located in the lower pole of the parotid gland, with a lymphoid node ~1.5 cm in diameter. The nodules of cases 3–7 were located in the superficial lobe of the parotid gland and were not well-defined within the parotid gland tissues. The nodule of case 7 was adhesive to the facial nerve. Inflammatory changes were observed around the nodule in one case. All the patients underwent a lump resection and partial parotidectomy. A total of five patients (cases 1, 2, 4, 5 and 8) were administered post-operative chemotherapy. The follow-up period ranged between 4 and 48 months. The lymphomas of cases 3 and 8 recurred, but no patients succumbed to their diseases.

### Pathological manifestation

The gross appearance of five of the tumor samples was solid and three were cystic. Cases 5–7 were light yellow in color, cases 1–3 were yellow and cases 4 and 8 were brown. The paraffin slice of case 4 displayed high hyperplasia of the lymphocytes and was identified in the right parotid gland. The lymphoma and partial small glands were broken by mononuclear B cells, and the patient was consequently diagnosed with MALT-lymphoma. Case 8 was diagnosed with MALT-lymphoma by combining tissue imaging with immunohistochemistry. Case 5 was diagnosed with bilateral lymphocytic mumps and MALT-lymphoma. Case 6 was diagnosed with benign lymphoepithelial lesions of the right parotid gland and MALT-lymphoma. Case 7 was diagnosed with benign lymphoepithelial lesions of the left parotid gland and lymphoid node reactive hyperplasia; a diagnosis of MALT-lymphoma was made through a consultation with the Peking Union Medical University following the recurrence of the disease. Cases 1–3 were diagnosed with MALT-lymphoma only. All the patients underwent immunohistochemical examinations (Table II).

### CT imaging features of MALT-lymphoma of the parotid gland

**Figure 1:**
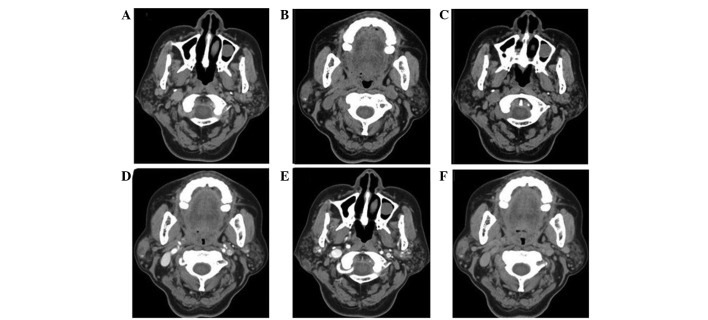
CT of a 68-year-old male patient showing the bilateral parotid gland with increased density and asymmetry. Two oval shaped, unclear foci with necrosis and calcification spots were observed on the right parotid gland. The necrosis was more apparent following CT enhancement. (A and B) Plain CT scan. (C and D) Arterial and (E and F) venous phases. CT, computed tomography.

**Figure 2:**
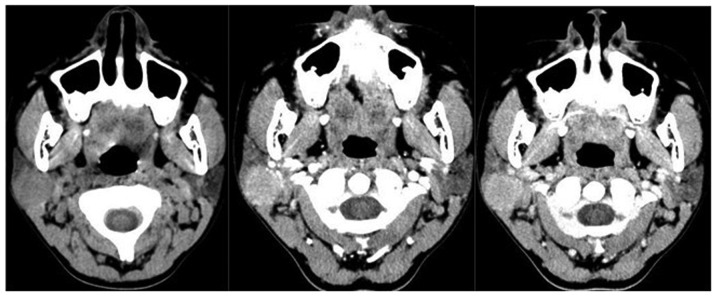
CT of a 14-year-old female patient showing a round soft tissue mass invading the deep lobe. The unclear margin between the sternocleidomastoid and the mass was more apparent following CT enhancement. CT, computed tomography.

**Figure 3:**
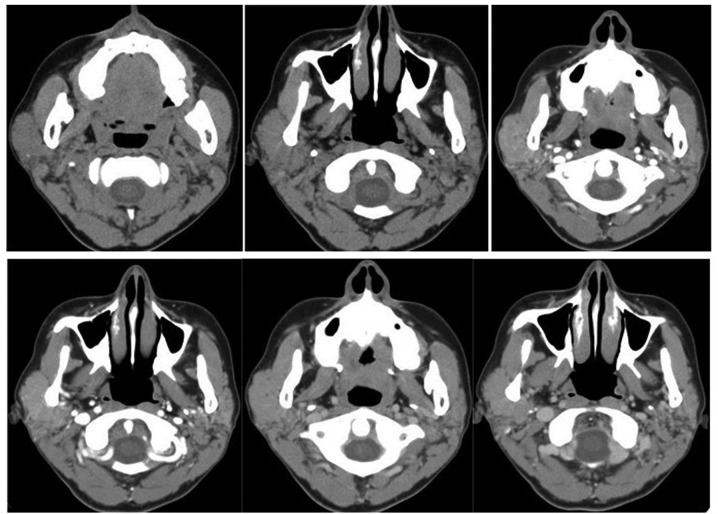
CT of a 58-year-old female patient showing two foci on the bilateral parotid gland, forcing the right side into an irregular shape, with slight lobulation. The necrosis inside the foci was more apparent following CT enhancement. The shape of the vessels was enhanced on the enhancement CT scan and the margin was unclear between the focus and the mass. CT, computed tomography.

As shown in Table II, all the patients were diagnosed with benign lesions. Two cases were also diagnosed with inflammation, three with mixed tumors, two with glandular lymphomas and one with a benign tumor. A total of 14 nodules were identified. Four cases were unilateral single lymphomas, one was a unilateral multiple lymphoma with two nodules, two were bilateral single lymphomas with four nodules and one case was a bilateral multiple lymphoma with four nodules (Table III). The sizes of the nodules ranged between 0.60 and 5.44 cm, with the majority (12 nodules) in the 0.5–3 cm range. The main bodies of the nodules were located in the superficial lobe. Only three nodules had invaded into the deep lobe. Two nodules were round and the remaining six were irregular in shape. Seven cases contained slight lobulation. The margins in seven cases were unclear and the masses of six cases were adhered to muscles. One case was cystic, four were with punctiform necrosis and the remaining three cases contained calcification. The solid section of the lesion appeared as an evenly dense soft tissue density on the CT and the range of the CT values was 35–59 HU. The solid area revealed a pattern of even enhancement, particularly in the arterial phase. The increased range of the CT value was 20–56 HU, mostly 20–40 HU. The increased range of the CT value of the venous phase was 18–34 HU compared with the plain CT scan. The density of the venous phase was slightly lower than that of the arterial phase. The decreased CT value of the venous phase ranged from −1–22 HU, often ≤15 HU, compared with the arterial phase. Two cases exhibited swelling of the parapharyngeal space lymphoid node. The density of the bilateral parotid glands was uneven in five cases and the structure was disordered. Multiple punctiform, nodular, striped slightly high density shadows were observed (Figs. 1, 2 and 3).

## Discussion

MALT-lymphoma, also called the marginal lymphoma, is a mild malignant tumor of the B lymphocytes that occurs outside the lymphoid nodes and accounts for 4–13% of all lymphoma (4–6). The most common site of occurrence is the gastrointestinal tract, accounting for 45–56% of all MALT-lymphomas. Other commonly occurring places include the lungs, eyes, conjunctiva, thyroid, parotid gland, skin and breasts (4). Primary lymphomas of the parotid gland form 0.6–5% of all the tumors of the parotid gland and tumor-like lesions. Marginal lymphomas of B lymphocytes form ~30% (7,8). Although MALT-lymphomas occur in structured lymph tissues, numerous MALT-lymphomas occur in organs or tissues without intrinsic MALT in normal conditions, including the stomach, salivary gland and thyroid. Chronic inflammation or autoimmune diseases are the foundation for these structures to acquire structured lymphoid tissues. Acquired lymphoid tissues are the premise of MALT-lymphomas (4). Li *et al* revealed that MALT-lymphomas were based on chronic inflammation, which may transfer for a long distance in the late period and undergo a transition process from reactive lymphoid hyperplasia to lymphoma of the B lymphocytes (9). Certain cases may transform into diffuse large B-cell lymphomas. MALT-lymphomas of the parotid gland have this pathological feature. In the present study, five cases demonstrated chronic inflammation and the infiltration of lymphocytes.

The pathological diagnostic standard of MALT-lymphoma combines paraffin slices with immunohistochemistry. The paraffin slice identifies the monoclonal property and the immunohistochemistry identifies the origin of the B lymphocytes. B cells express CD20 and CD79a. T cells are negative for CD5, CD10, CD23 and CD43. The surface immunoglobulins are IgM^+^, Bcl-2^+^ and IgD^−^ (10). In the present study, the cases were all positive for CD20 and CD79a and negative for CD10. The majority of the cases were negative for CD3, CD5 and CD43. Two cases were weakly-positive for CD3, CD5 and CD43, which may be associated with the reactive T-cell hyperplasia that was observed around the lesion.

The patients that are affected by MALT-lymphoma are usually adults with a median age of 60 years and a male:female ratio of 1.2:1 (11). In the present study, the male:female ratio was1:7, which was affected by the number of cases. The median age was 52 years, which was almost consistent with the literature, however one patient was 14 years old. MALT-lymphoma may occur unilaterally or bilaterally. In the present study, five cases were unilateral and three were bilateral. The course of the disease varied between two months and six years. The main clinical presentation was a painless lump and no apparent constitutional symptoms were observed. Symptoms of a dry mouth and eyes were observed, similar to the symptoms of Sjögre’s syndrome. One case demonstrated mild facial nerve injury symptoms. Additionally, the boundary between the lump and the surrounding tissues may be unclear in MALT-lymphoma. In the present study, the lumps of six cases were not separate from the surrounding tissues and exhibited the clinical features of malignant tumors. The lumps were of moderate hardness and could only be moved slightly if at all.

The parotid gland contains MALT in normal conditions. B lymphocytes infiltrate and assemble within the parotid gland during autoimmune disease, which increases the succeptibility to MALT-lymphomas. Kassan *et al* (12) believed that patients with Sjögre’s syndrome were 44 times more likely to develop MALT-lymphomas compared with normal individuals. Wang *et al* (13) revealed that Sjögre’s syndrome is associated with the occurrence, development and clinical performance of non-Hodgkin lymphomas of the parotid gland. Certain patients with Sjögre’s syndrome may develop lymphomas. In the present study, seven cases had apparent Sjögre’s syndrome symptoms, including a dry mouth and eyes, which was consistent with the literature. As a result, strong vigilance is required to avoid MALT-lymphoma in patients with Sjögre’s syndrome and lumps in the parotid gland.

Imaging studies with regard to MALT-lymphomas of the salivary gland have not been previously observed in the literature. The present study identified that MALT-lymphomas often occur unilaterally and that they may also occur bilaterally. Wen *et al* (14) reported nine cases of MALT-lymphoma of the salivary gland, one of which was a bilateral multiple case. Li *et al* (15) reported three cases of MALT-lymphoma of the parotid gland and one was a bilateral multiple case. The size of the nodule is often 0.5–3 cm and is commonly located in the superficial lobe of the parotid gland. The shape of the nodule may occasionally be round, but is often irregular. The margin may be clear or the nodule may be attached to the surrounding muscles with an unclear margin. The nodule shares common features with malignant tumors. MALT-lymphoma of the parotid gland is a slightly malignant tumor. Therefore, the imaging performance is between that of typical benign tumors and of malignant tumors. In the present study, the soft tissue density was evenly dense and of slightly higher density than the surrounding muscles on the plain scan. The CT value was ~40 HU, although four cases reached >50 HU. Certain nodules displayed necrosis and calcification and the CT value on the plain scan was slightly higher than that of other parotid gland tumors compared with previous studies, which may be associated with the intensive arrangement of the tumor cells (9,14,16). Following enhancement, the CT scan revealed a pattern of even enhancement, with the exception of the necrotic and cystic regions. The CT value of the arterial phase increased by 20–56 HU, while that of the venous phase increased by 18–34 HU. The degree of enhancement of the venous phase was slightly lower than that of the arterial phase. The density of the venous phase was slightly higher than that of the arterial phase in one case. The general enhancement pattern was almost consistent with that of MALT-lymphomas of other organs (2,3,15). Extranodal lymphomas originate from the organ mesenchyme and the tumor often grows along the edge of the organ, thus structure residues of the primary tissue are commonly observed in tumors. In the present study, the density of the majority of the tumors was even. Certain tumors were not even due to the residues of the primary tissue, including those of the organic intrinsic vessels, the intermuscular space and the renal cortex. The degree of enhancement is even and often ranges from low to moderate, with the exception of cerebral tumors. The arterial phase shows a pattern of slight enhancement, while the venous phase shows a pattern of moderate enhancement or dynamic enhancement (16). The cases in the present study are relatively consistent with the literature. The lymphomas were mostly located in the envelop of the superficial lobe of the parotid gland and demonstrated an even density on the plain scan or the enhancement, with the exception of the necrotic regions. However, in these cases, the arterial phase revealed a pattern of enhancement, which may be associated with the delayed time.

The present study identified that the MALT-lymphomas of the parotid gland demonstrated even isodense nodules whose densities were slightly higher than that of the surrounding muscles and other tumors of the parotid gland on plain CT scan. The lymphomas easily became attached to the surrounding muscles. The background of the parotid gland often showed inflammation of heterogeneous density and a pattern of moderate enhancement, with no significant features on the imaging. It is necessary to distinguish MALT-lymphoma from benign or malignant tumors.

The mixed tumors of the parotid gland were the most common benign tumors of the parotid gland identified. The CT performance was the closest to that of MALT-lymphoma as it revealed even or uneven density nodules of the soft tissue on the CT. The nodules were nearly-round with clear margins and the density was slightly lower than that of MALT-lymphoma on the plain scan and revealed a pattern of slightly delayed enhancement. The amplitude of the mixed tumor was weak in the early enhancement phase and the mixed tumor had certain differences compared with MALT-lymphoma in the moderate enhancement, which may provide a reference for identification.

Glandular lymphomas of the parotid gland, basal cytoma and myoepithelioma are another three common benign tumors, which contain round or near-round nodules with clear margins on the plain scan, often with cystic necrosis (10). In the present study, the glandular lymphomas showed a pattern of early rapid enhancement with rapid clearance of the contrast agent, while the basal cytoma and myoepithelioma showed a pattern of continuous significant enhancement. Glandular lymphomas may be bilateral and multiple. The affected patients in the present study had a clinical history of long-term smoking. Therefore, it was not difficult to distinguish glandular lymphoma cases from those of MALT-lymphoma.

Primary epithelioid malignant tumors of the parotid gland were complex and their CT performances were varied, with typical features of large nodules, inner necrosis, unclear margins, adhesion to the surrounding tissues and apparent uneven enhancement. It was relatively easy to identify the tumors with typical malignant features, but it was difficult to identify other relatively small and indistinctive nodules. With the exception of the imaging performance, the most significant basis for diagnosis was whether there were the symptoms of a dry mouth and eyes, as in Sjögre’s syndrome.

The treatment of MALT-lymphomas of the parotid gland involves the surgical excision of the nodule and the gland, with post-operative radiotherapy or chemotherapy. Zinzani *et al* (4) reported that the recurrence rate for MALT-lymphomas may be up to 17%. Recurrence includes the transfer of local or surrounding lymphoid nodes and responds well to treatment with chemotherapy assisted by a local injection of interferon-α-II. In the present study, two cases recurred, one of which was not administered post-operative chemotherapy. Six cases did not experience recurrence.

The patients with MALT-lymphoma of the parotid gland in the present study were all diagnosed with benign tumors pre-operatively, mainly due to the fact that the CT performance was similar to that of benign tumors and the cognition of its imaging performance was lacking. MALT-lymphomas of the parotid gland have certain features, including similar clinical symptoms to Sjögre’s syndrome, and commonly occur in females. The condition frequently appears in middle-aged and elderly patients and is often distributed in the superficial lobes, but may also invade into the deep lobes and be present as bilateral multiple lymphoma. The uniform hyperdensity is higher than that of the surrounding muscle tissues on the plain CT scan, and occasionally calcification and necrosis is observed. The nodules may be of irregular morphology and often appear to be slight lobulated with unclear margins and attached to the surrounding muscles. The parenchyma shows moderate enhancement and the degree of enhancement of the arterial phase is close to that of the venous phase. These features may aid the imaging diagnosis.

## Figures and Tables

**Table I:** Clinical characteristics in eight cases.

Case	Gender	Age, years	Sjögre’s syndrome	Facial nerve involvement	Range of motion	Malignant neck lymph nodes	Treatment	Recurrence	Follow-up time, months
1	F	62	Y	Y	Poor	N	Surgery and chemoradiotherapy	N	48
2	M	14	Y	N	Poor	N	Surgery and chemoradiotherapy	N	24
3	M	80	Y	N	Good	Y	Surgery	Y	14
4	M	58	Y	N	Good	N	Surgery and chemoradiotherapy	N	14
5	M	46	N	N	Good	N	Surgery and chemoradiotherapy	N	19
6	M	38	Y	N	Medium	N	Surgery	N	19
7	M	68	Y	N	Medium	N	Surgery	N	4
8	M	42	Y	N	Medium	N	Surgery and chemoradiotherapy	Y	9

F, female; M, male; Y, yes; N, no.

**Table II:** Immunohistochemistry in eight cases.

		Immunohistochemistry					
		
Case	Tumor pathological type	CD3	CD5	CD10	CD20	CD43	CD79a
1	MALT-L	−	−	−	+	−	+
2	MALT-L	−	−	−	+	−	+
3	MALT-L	−	−	−	++	−	+
4	MALT-L	−/+	−	−	+	−	+
5	Lymphoblastic parotitis with MALT-L	−	−	−	+	−	+
6	Benign lymphoepithelial lesion with MALT-L	−	−	−	+	+	+
7	Parotid MALT-L	+	+	−	+	−	+
8	MALT-L	+	+	−	++	+	+

MALT-L, mucosa-associated lymphoid tissue lymphoma; −, negative; −/+, weak positive; +, positive; ++, strong positive.

**Table III:** CT characteristics in eight cases

Case	Side	Number	Size, cm (width × length)	Location	Morphology	Lobular	Margin
1	Unilateral	1	1.42×2.49	Deep	Irregular	Yes	Unclear
2	Unilateral	1	2.57×2.84	Deep	Round	Yes	Unclear
3	Unilateral	1	0.96×1.13	Superficial	Round	No	Clear
4	Unilateral	1	0.95×2.42	Superficial	Irregular	Yes	Unclear
5	Bilateral	4	0.68×1.27, 0.60×1.00 0.60×0.60, 0.80×1.00	Superficial	Irregular	No	Unclear
6	Bilateral	2	1.96×3.52, 0.64×0.88	Superficial	Irregular	Yes	Unclear
7	Unilateral	2	1.57×2.58, 1.26×1.99	Superficial	Irregular	Yes	Unclear
8	Bilateral	2	2.51×5.44, 1.23×1.28	Deep	Irregular	Yes	Unclear

CT, computed tomography.
